# Preliminary Phytochemical Screening and *In Vitro* Antioxidant Activities of *Parkinsonia aculeata* Linn.

**DOI:** 10.1155/2014/756184

**Published:** 2014-04-13

**Authors:** Sonia Sharma, Adarsh Pal Vig

**Affiliations:** Department of Botanical and Environmental Sciences, Guru Nanak Dev University, Amritsar, Punjab, India

## Abstract

Butanol and hexane leaves extracts of *Parkinsonia aculeata* L. (Fabaceae) were assessed for its antioxidant potential by *in vitro* methods. Phytochemical analysis and antioxidant activity of plant extracts were studied using different *in vitro* assays. UPLC analysis of extracts was carried out for the identification of chemical constituents. The total phenolic contents of the butanol and hexane leaf extract were 42 mgGAE/g and 34 mgGAE/g whereas flavonoid contents of these extracts were found to be 0.044 mgRE/g and 0.005 mgRE/g, respectively. Among both extracts, butanol extract shows maximum inhibition (%) of 93.88%, 80.02%, 52.06%, 94.68%, and 69.37% in DPPH, non-site-specific and site-specific, FTC, and TBA assays and absorbance of 0.852 and 0.522 in reducing power and CUPRAC assay at the highest concentration tested. The FRAP and TAC values of butanol extract were found to be 678 **μ**M Fe(II)/g and 36 mgAAE/100 mg. UPLC analysis of extracts revealed the presence of various polyphenols. The tested plant extracts were found to possess potent antioxidant and free radical scavenging activity which may be due to the presence of flavonoids and polyphenols.

## 1. Introduction

Oxidative stress induced by reactive oxygen species (ROS) is implicated in the pathogenesis of a variety of diseases such as cancer [1], atherosclerosis [2], hypertension, and coronary artery diseases. The tissue injury caused by ROS includes lipids, protein, and DNA damages and oxidation of important enzymes [3]. The intake of antioxidants such as flavonoids and polyphenols has been effective in the prevention of these diseases [4]. Among natural antioxidants, phenolic compounds are known to quench oxygen-derived free radicals by donating a hydrogen atom or an electron to the free radical [5] and they have shown to be neutralizing free radicals in various models system [6]. Studies have shown that these antioxidant compounds possess antimutagenic, antitumor, anticarcinogenic, and antiviral activities [7]. The ingestion of natural antioxidants has been associated with reduced risks of cancer, cardiovascular disease, diabetes, and other diseases associated with ageing [8] and, in recent years, there has been a worldwide trend towards the use of the natural phytochemical present in berry crops, herbs, oilseeds, fruits, and vegetables, and so forth [9].

*Parkinsonia aculeata* is a small, spiny deciduous tree, grown up to 5–10 m high and trunk up to 40 cm in diameter. It remains green throughout the year and appears leafless after leaflets falls. Leaf, fruit, and stems are taken orally to treat malaria and fever and as an abortifacient. Flower and leaf extraction in alcohol are used to treat rheumatism. However the beneficial effects of these* P. aculeata* extracts have not been investigated and are largely overlooked at the biochemical and biological levels. The aim of the present study was to evaluate the phytochemical analysis, antioxidant activities, free radical scavenging activity, and reducing power of the extracts of* P. aculeata* and to evaluate which properties contribute to this effect. Leaves of the plant have been reported to contain C-glycosyl flavones like orientin, vitexin, and iso vitexin [10].

## 2. Materials and Methods

### 2.1. Chemical Reagents

Folin-Ciocalteu reagent, sodium carbonate, gallic acid, rutin, aluminium chloride, sodium nitrate, sodium hydroxide, 2,2-diphenyl-1-picrylhydrazyl (DPPH), trichloroacetic acid, potassium ferricyanide, sodium acetate buffer, neocuproine, deoxyribose, EDTA, potassium phosphate buffer, hydrogen peroxide, ascorbic acid, TBA, 2,4,6-tripyridyl-s-triazine (TPTZ), ferric chloride, HCl, ammonium molybdate, sodium phosphate, sulphuric acid, ammonium thiocyanate, and all other chemicals used were of analytical grade.

### 2.2. Preparation of Plant Extracts

The leaves of* P. aculeata* were collected in the month of July from the tree growing near Guru Nanak Dev University (Punjab, India). Botanical identification was made from Herbarium of Department of Botanical & Environmental Sciences, GNDU, where a voucher of specimen (accession number 6774, dated: June 17, 2012) was deposited. The plant sample was ground to fine powder and precisely weighed amount of the powder was extracted with butanol and hexane solvents and was vaccum dried with Buchi Rotavapor to obtain the dried butanol and hexane extract. These extracts were used for the phytochemical analysis and determination of antioxidant activities and total phenolic and flavonoid contents.

### 2.3. Phytochemical Analysis

The dry extracts of* P. aculeata* were subjected to phytochemical tests for compounds which include tannins, flavonoids, alkaloids, saponins, and so forth in accordance with the methods of Chakraborty et al. [11] with little modifications.

### 2.4. Determination of Total Phenolic Content

Total phenolic content was determined using the Folin-Ciocalteu reagent [12]. To 100 *μ*L of extract and 0.5 mL of Folin-Ciocalteu reagent were added, and the mixture was allowed to stand for 6 min, before adding 1.5 mL of 20% Na_2_CO_3_ solution. The total volume of solution was adjusted to 5 mL with distilled water. After 2 h of incubation at room temperature, the absorbance at 765 nm was measured in triplicate. Gallic acid (0–200 *μ*g/mL) was used for calibration of standard curve. The results were expressed as milligram gallic acid equivalent (mgGAE)/g dry weight of plant material.

### 2.5. Determination of Total Flavonoid Content

The method given by Zhishen et al. [13] was used for analyzing total flavonoid content (TFC) employing rutin as a standard. In 1 mL of extract, distilled water (dH_2_O), 5% NaNO_2_, and 10% AlCl_3_ were added. After 5 min incubation, 1 M NaOH was added followed by the addition of 2.4 mL dH_2_O to get the final volume of 10 mL. The absorbance of extract and standard were taken at 510 nm by US-VIS spectrophotometer. The total flavonoid content was expressed as mg rutin equivalents per gram (mg RE/g) through the calibration curve with rutin.

### 2.6. Antioxidant Activity

#### 2.6.1. DPPH-Radical Scavenging Activity

The hydrogen atom donating ability of the different plant extracts was determined from the decolorization of purple colored methanol solution of 2,2-diphenyl-1-picrylhydrazyl (DPPH) [14]. In this assay, 200 *μ*L of extract solution and reference compound was mixed with 3 mL of 0.1 M of DPPH solution in methanol. After 30 min, absorbance was measured at 515 nm and gallic acid was used as a reference compound. The decrease in absorption was correlated with the radical scavenging activities (percent inhibition) of samples. The percentage of inhibition was calculated by the following:

(1) $$\begin{array}{c} \%\;\;\text{antioxidant}\;\text{activity}=\left[\frac{\left(A_{c}-A_{s}\right)}{A_{c}}\right]\times 100, \end{array}$$

where *A*_*c*_ is absorbance of control; *A*_*s*_ is absorbance of sample.

#### 2.6.2. Reducing Power Assay

The reducing power of the extracts of* P. aculeata* was determined according to the method of Oyaizu [15]. Various concentrations of butanol and hexane extracts and standard (1 mL) were mixed with 200 *μ*M potassium phosphate buffer (pH 6.6) and 1% of potassium ferricyanide. After 20 min incubation at 50°C, an aliquot of 10% trichloroacetic acid was added to the mixture. The mixture was centrifuged at 3000 g for 10 min and, after that, upper layer was mixed with dH_2_O and 0.1% of ferric chloride, and the absorbance was measured at 700 nm. The assays were carried out in triplicate and the results are expressed as mean ± standard error (SE). Increase in absorbance of sample with concentrations indicates high reducing potential of the samples.

#### 2.6.3. Cupric Ions Reducing Assay (CUPRAC)

Cupric ions can damage the central nervous system and endocrine. In order to determine the cupric ions (Cu^2+^) reducing ability of butanol and hexane extracts of* P. aculeata*, the method proposed by Apak et al. [16] was used. For this reason, 0.01 M of CuCl_2_ solution, 7.5 mM of ethanol neocuproine solution, and 1.0 M of CH_3_COONH_4 _buffer solution were added to each test tube, respectively. Then, different concentrations of standard antioxidant (gallic acid) and extracts were added to the each tube, separately. Finally, total volume was adjusted to 2 mL with dH_2_O and mixed well. Tubes were kept at room temperature for 30 min. Absorbance was measured at 450 nm against a reagent blank. Increased absorbance of the reaction mixture indicates increased reduction capability of solution.

#### 2.6.4. Non-Site-Specific Hydroxyl Radical Scavenging Activity

Non-site-specific hydroxyl radical scavenging activity of extracts was measured according to the method of Halliwell et al. [17]. 1 mL of Haber Weiss reaction mixture consists of 2-deoxyribose (10 mM), Fe(III) chloride (10 mM), 1 mM EDTA, and 10 mM of H_2_O_2_ with or without extract or standard in potassium phosphate buffer (pH 7.4). The reaction was triggered by adding ascorbic acid (1 mM) which reduces Fe^3+^ to Fe^2+^ ions. The reaction mixture was incubated for 1 h at 37°C and further heated in a boiling water bath for 20 min. To the above solution, 0.5% of TBA and 10% of TCA were added. The pink colour development was measured at 532 nm against a blank containing phosphate buffer.

#### 2.6.5. Site-Specific Hydroxyl Radical Scavenging Activity

This procedure is similar to that used to measure the non-site-specific hydroxyl radical scavenging activity. In this assay, EDTA was replaced by a similar volume of potassium phosphate buffer [18].

The inhibitory effect of extract or reference was calculated as

(2) $$\begin{array}{c} \%\;\;\text{Hydroxyl}\;\text{radical}\;\text{scavenging}\;\text{capacity}=\left(\frac{1-A_{s}}{A_{c}}\right)\times 100. \end{array}$$

Here *A*_*c*_ is absorbance of control; *A*_*s*_ is absorbance of sample solution.

#### 2.6.6. Ferric Reducing Antioxidant Power (FRAP)

Reducing power of both the extracts (butanol and hexane) of* P. aculeata* was done according to Benzie and Strain [19] with some modifications. The stock solutions contain 300 mM acetate buffer (3.1 g C_2_H_3_NaO_2_-3H_2_O and 16 mL C_2_H_4_O_2_), pH 3.6, 10 mM TPTZ (2,4,6-tripyridyl-s-triazine) solution in 40 mM HCl, and 20 mM FeCl_3_ 6H_2_O solution. The fresh working solution was prepared by mixing TPTZ solution, FeCl_3_ 6H_2_O solution, and acetate buffer in the ratio of 1 : 1 : 10 and it was warmed at 37°C for 25 min before use. Plant extract or reference was allowed to react with FRAP solution in the dark condition for 30 min. Readings of the colored product (ferrous tripyridyltriazine complex) were then measured at 593 nm. The standard curve was linear between 100 and 1000 *μ*M FeSO_4_. Results are expressed in *μ*M (Fe(II)/g) dry mass. Decreased absorbance indicates ferric reducing power capability of sample.

#### 2.6.7. Total Antioxidant Capacity

The antioxidant activity of butanol and hexane extracts was evaluated by the transformation of Mo (VI) to Mo (V) to form phosphomolybdenum complex [20]. 0.2 mL of extracts was combined with 2 mL of reagent solution (0.6 M sulphuric acid, 28 mM sodium phosphate, and 4 mM ammonium molybdate). The test tubes were capped with foil and incubated in water bath at 95°C for 95 min. After cooling to room temperature, the absorbance of each solution was measured at 695 nm against blank.

#### 2.6.8. Ferric Thiocyanate (FTC) Method

The standard method described by Kikuzaki et al. [21] was used for ferric thiocyanate determination. A mixture of 4 mg of sample in 4 mL of 99.5% ethanol, 4.1 mL of 2.51% linoleic acid in 99.5% ethanol, 8.0 mL of 0.02 M phosphate buffer (pH 7.0), and 3.9 mL of distilled water contained in covered test tubes was placed in an oven at 40°C in the dark. After that, 0.1 mL of the reaction mixture from the above solution was transferred to a test tube and 9.7 mL of 75% aqueous ethanol followed by 0.1 mL of 30% aqueous ammonium thiocyanate and 0.1 mL of 0.02 M ferrous chloride in 3.5% hydrochloric acid were added to it. Three minutes after the addition of ferrous chloride to the reaction mixture, the absorbance of the resulting mixture of red color was measured at 500 nm after every 24 h until the absorbance of the control reached its maximum value. Gallic acid (final concentration of 0.02% w/v) was used as positive control, while the negative control used was the mixture without the plant extract.

#### 2.6.9. Thiobarbituric Acid (TBA) Method

The method of Kikuzaki and Nakatani [22] was used for the determination of free radicals present in the butanol and hexane leaf extracts. The final sample concentration from 4 mg in 4 mL of ethanol from the same samples prepared for FTC assay was used. 2 mL of 20% trichloroacetic acid and 2 mL of 0.67% of thiobarbituric acid were added to 1 mL of sample solution from the FTC method. The mixture was placed for 10 min in a boiling water bath and then centrifuged after cooling at 3000 rpm for 20 min. The absorbance activity of the supernatant was measured at 552 nm and recorded after it has reached its maximum value.

#### 2.6.10. Statistical Analysis

Each experiment was performed in triplicate and results were recorded as mean ± standard error (SE).

#### 2.6.11. Ultra Performance Liquid Chromatography (UPLC)

Butanol and Hexane extracts were subjected to UPLC in order to identify the presence of various polyphenolic compounds like gallic acid, epicatechin, umbelliferone, coumaric acid, and so forth. The preparation of sample to identify active constituents is as mentioned below.

*Sample Preparation*. For UPLC analysis, the dried butanol and hexane extracts were dissolved in methanol (HPLC grade) and filtered through 0.22 *μ*m syringe filter. Plant samples were analyzed on Shimadzu UPLC Nexera system (Schimadzu, USA) with photodiode array detector. C_18_ column (150 mm × 4.6 mm) is used at 25°C temperature. Mobile phase used for analysis consisted of solution I (0.1% acetic acid) and solution II (methanol). Flow rate was 1 mL/min and injection volume was 5 *μ*L. Content of peaks was determined using software provided with Shimadzu UPLC Nexera system (USA).

## 3. Results

### 3.1. Phytochemical Screening

The phytochemical analysis conducted on butanol and hexane leaf extracts of* P. aculeata* revealed the presence of alkaloid, carbohydrate, glycoside, saponin, protein and amino acids, phenolics, and flavonoids (Table 1). The total phenolic content of butanol and hexane leaf extracts was 42 mg GAE/g and 34 mg GAE/g (*y* = 0.001*x* + 0.034, *R* ^2^ = 0.990). The total flavonoid content of butanol and hexane extracts of plant was 0.044 mgRE/g and 0.005 mgRE/g, respectively, with reference to standard curve.

### 3.2. DPPH Assay

**Figure 1:**
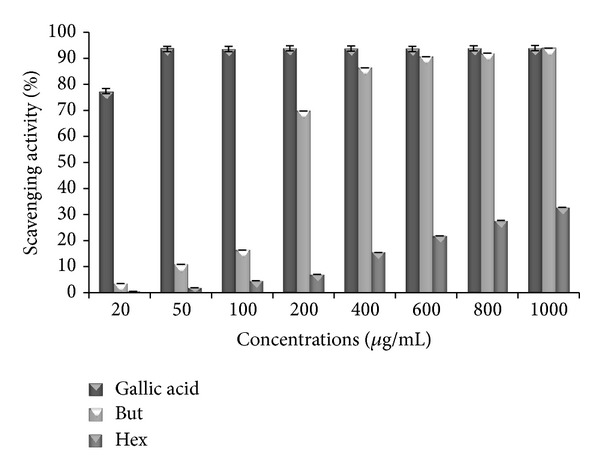
DPPH scavenging activity of standard (gallic acid) and extracts of* P. aculeata*. Data are represented as mean ± SE (*n* = 3). But: butanol extract; Hex: hexane extract.

A dose-response relationship was observed for both butanol and hexane extracts (Figure 1). Butanol extract demonstrated the strongest percentage inhibition and radical scavenging activity as compared to hexane extract. Hexane extract shows percentage inhibition of 32.71% which is less than that of butanol. The butanol extract appeared to be as potent as gallic acid with a maximum inhibition of 93.88% at 1000 *μ*g/mL which is nearly potent as compared to 93.89% for gallic acid at same concentration.

### 3.3. Reducing Power Assay

**Figure 2:**
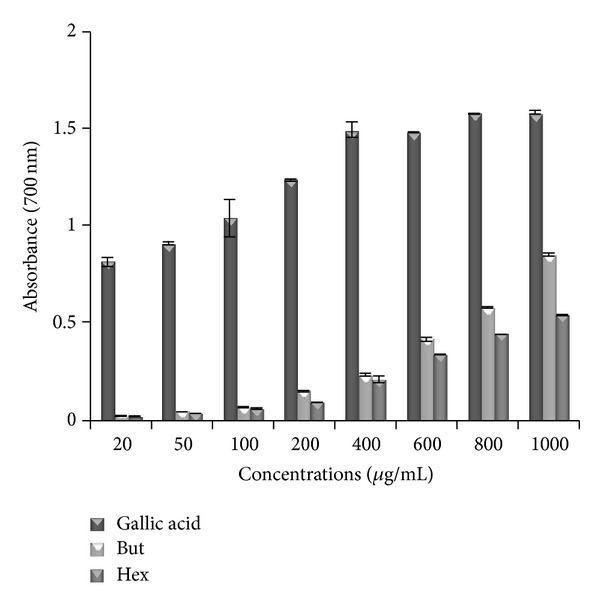
The reductive ability of* P. aculeata *extracts and standard gallic acid. The absorbance was plotted against concentrations of samples. All values represented mean ± SE (*n* = 3). But: butanol extract; Hex: hexane extract.

In this study, the reducing power of both butanol and hexane leaf extract of* P. aculeata* increased with concentration. Among the butanol and hexane extracts, butanol extract shows high absorbance, that is, 0.852 ± 0.008, as compared to absorbance, that is, 0.536 ± 0.003, of hexane extract, respectively, at the highest concentration of 1000 *μ*g/mL (Figure 2). The high reducing power of butanol extract may be due to its higher phenolic and flavonoid content as compared to hexane solution.

### 3.4. CUPRAC Assay

**Figure 3:**
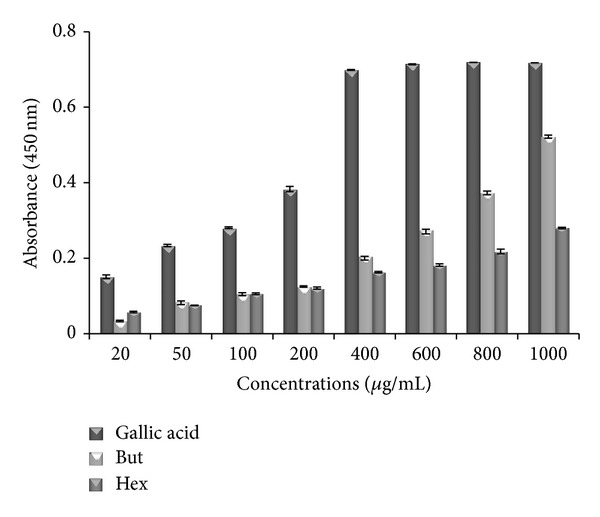
Reducing power of butanol (But) and hexane (Hex) extracts of* P. aculeata *and standard antioxidant (gallic acid) by CUPRAC assay. Values represented mean ± S.E (*n* = 3).

CUPRAC (cupric reducing antioxidant) assay has been used by many researchers to determine reducing power of different test solutions. In this study, both butanol and hexane leaf extracts of* P. aculeata* show increase in absorbance with increase in concentration. Among both extracts butanol shows high absorbance then hexane extract. Maximum absorbance showed by butanol and hexane is 0.522 ± 0.004 and 0.28 ± 0.002 at higher concentration of 1000 *μ*g/mL. At this higher concentration, standard, that is, gallic acid, shows absorbance of 0.718 (Figure 3).

### 3.5. Non-Site-Specific and Site-Specific Hydroxyl Radical Scavenging Activity

**Figure 4:**
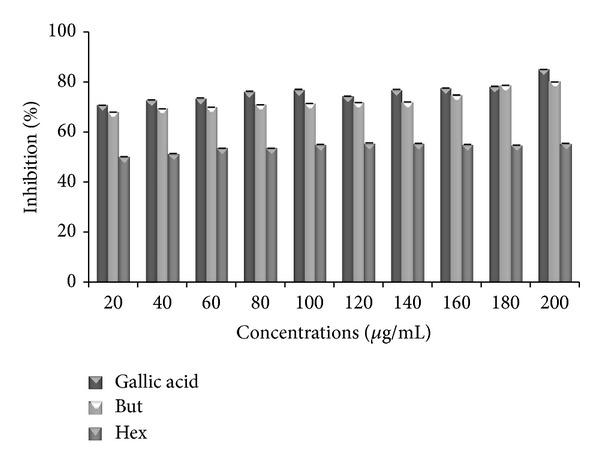
Antioxidant effects of hydroxyl radical (-EDTA) in butanol (But) and hexane (Hex) extracts of* P. aculeata. *Data are represented as mean ± SE (*n* = 3).

**Figure 5:**
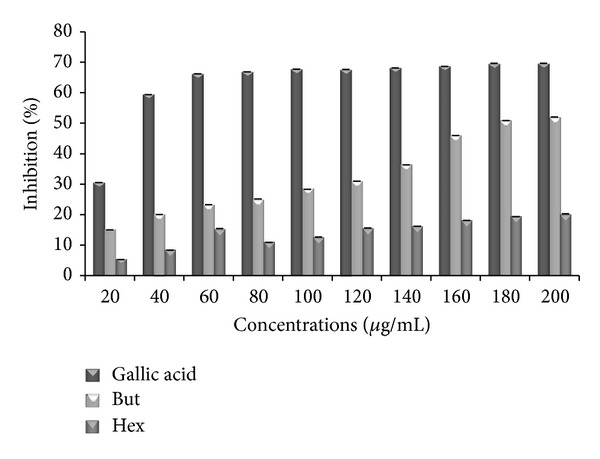
The scavenging activity of hydroxyl radical in standard (gallic acid) and butanol (But) and hexane (Hex) extracts of* P. aculeata. *Data are represented as mean ± SE (*n* = 3).

The results showed that concentration dependent inhibition of extracts and standard against hydroxyl radical-induced degradation of deoxyribose was observed in both non-site-specific and site-specific hydroxyl assays (Figures 4 and 5). The results revealed that butanol extract is the most effective inhibitor of free radical and show a remarkable scavenging potential of 80.02% at 200 *μ*g/mL in non-site-specific assay whereas in site-specific, it showed 52.06% inhibition at the same concentration. However, hexane extract exhibited percent inhibition of 50.5% and 20.31% in non-site-specific and site-specific assays at 200 *μ*g/mL. At the same concentration, the percentage inhibition values were 85.005% and 69.68% for gallic acid in non-site-specific and site-specific assays.

### 3.6. FRAP

In this assay the ability of butanol and hexane leaf extract of* P. aculeata* to reduce ferric ions was determined. FRAP assay measures the changes in absorbance at 593 nm of blue coloured Fe^+2^-tripyridyltriazine solution from colourless Fe^+3^ form by the action of electron donating antioxidant. To calculate the FRAP value (*μ*M Fe(II)/g) for extracts, a linear regression equation of the standard (FeSO_4_·7H_2_O) was plotted. The FRAP value of butanol extract, that is, 678 *μ*M Fe(II)/g, is higher than the frap value, that is, 60 *μ*M Fe(II)/g, of hexane extract. A higher value indicates a higher ferric reducing power (Table 2).

### 3.7. Total Antioxidant Capacity

It was found that both butanol and hexane leaf extract of* P. aculeata* has ability to reduce Mo(VI) to Mo(V) by donating electron at acidic pH and leads to the formation of green phosphate. The reduction ability of butanol and hexane extract was found to be 36 mgAAE/100 mg and 0.75 mgAAE/100 mg dry weight of extract (Table 2). These values were calculated from the regression equation obtained for ascorbic acid.

### 3.8. FTC and TBA

**Figure 6:**
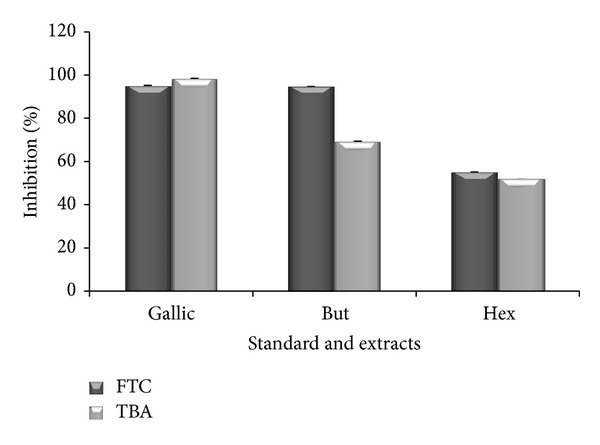
Antioxidant properties of standard (gallic acid) and leaves extracts of* P. aculeata*, determined by FTC and TBA method on sixth day of experiment at the highest concentration (1000 *μ*g/mL). FTC: ferric thiocyanate; TBA: thiobarbituric acid; But: butanol; Hex: hexane extract.

In FTC method, among both leaf extracts, butanol extract exhibited strong antioxidant potential with percent inhibition of 94.68%, whereas hexane extract shows 55.49% of inhibition at the highest concentration of 1000 *μ*g/mL whereas in TBA method, butanol and hexane extract showed 69.37% and 51.71% inhibition at the same concentration (Figure 6). Standard, that is, gallic acid, showed 95.54% and 98.46% in FTC and TBA method.

### 3.9. UPLC Analysis

**Figure 7:**
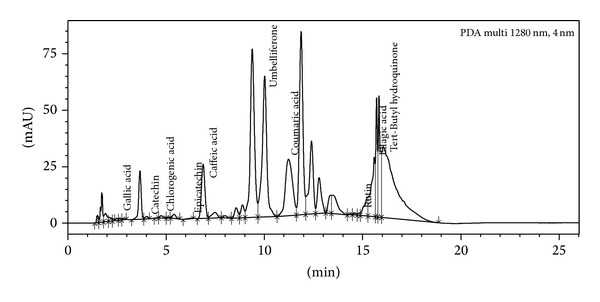
UPLC chromatogram of butanol extract showing the presence of different polyphenols.

**Figure 8:**
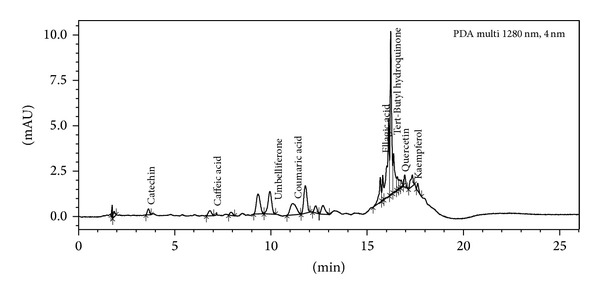
Chromatograph of hexane extract of* P. aculeata* leaves.

Figures 7 and 8 depict the presence of polyphenolic compounds in different extracts of leaves of* P. aculeata*. On the basis of chromatogram obtained for the butanol extract; it was concluded that leaves contain gallic acid, catechin, chlorogenic acid, epicatechin, caffeic acid, umbelliferone, coumaric acid, rutin, ellagic acid, and tert-Butyl hydroquinone. Chromatogram (Figure 8) of hexane extract shows the presence of catechin, caffeic acid, umbelliferone, coumaric acid, ellagic acid, tert-Butyl hydroquinone, quercetin, and kaempferol.

## 4. Discussion

Free radicals are constantly generated in living beings and can cause maximum damage to biomolecules leading to different types of disease. Synthetic drugs are protected against damage but they have various types of side effects. An alternative solution of this problem is to produce natural antioxidants from plant products. Many phytochemical compounds are known to support bioactive activities and thus responsible for the antioxidant. When antioxidant potential of synthetic antioxidant was compare with different extracts of leaf of* P. aculeata*, it also exerted strong antioxidant activity.

The presence of phenolic and flavonoid content in the leaf extract of this plant was earlier reported by Mruthunjaya and Hukkeri [23] and Singh et al. [24]. DPPH is a stable free radical, widely used to test the ability of plant extract to act as free radical scavenger and for the screening of presence of antioxidant activity of plant extract [25]. The degree of reduction in absorbance measurement is indicative of the radical scavenging potential of the extract. Results show that plant extracts show better antioxidant property as shown by standard. Previously, Singh et al. [24] reported that* P. aculeata *ethanol and ethyl acetate extract exhibited percentage inhibition of 86.7% and 75.29% at 0.5 mg/mL.

The capacity of compound to reduce Fe^3+^/ferricyanide complex to the Fe^2+^/ferrous form acts as an indicator of its potential antioxidant activity [26]. In this assay, the yellow colour of the FeCl_3_/K_3_Fe(CN)_6_ present in the solution changes to various shades of green and blue, depending on the reducing power of test solution. Therefore, measuring the greater absorbance of blue colour solution at 700 nm indicates greater reducing power [27]. Among both extracts butanol is good reducing agent as gallic acid. Similar increase in reducing power with increase in concentration of test solution was observed in different studies [28, 29].

In CUPRAC method, the main oxidizing agent is bis(neocuproine)copper(II)chloride(Cu(II)-Nc), which reacts with *n*-electron reductant antioxidants (AO). This reaction occurs in the following manner:

(3) nCu(Nc)22++n-electron  reductant  (AO) ⟶nCu(Nc)2++n-electron  oxidized  product+nH+.

So in this reaction Cu(II)-Nc is reduced to the colored Cu(I)-Nc chelate showing maximum absorption at 450 nm. In this assay, a higher absorption indicates high reducing power of cupric ions. The present study proved that butanol extract has high potent antioxidant activity as that of gallic acid (Figure 3).

Non-site-specific and site-specific hydroxyl radical scavenging assays show the antioxidant activity of butanol and hexane extracts and standard, that is, gallic acid, to inhibit hydroxyl radical mediated degradation of deoxyribose in an Fe^3+^-EDTA-ascorbic acid and H_2_O_2_ reaction solution. Hydroxyl radicals are the major active oxygen species causing lipid peroxidation and biological damage. The results so obtained indicated that butanol has strong affinity to bind to deoxyribose as compared to Fe^3+^ ion and so it protects the molecule of deoxyribose from radical damage caused by hydroxyl (OH).

The FRAP assay was shown to be a useful tool for determining antioxidant potential of solutions [30]. The main reaction behind this assay is as follows:

(4) $$\begin{array}{c} {\text{K}}_{3}\text{Fe}{\left(\text{CN}\right)}_{6}+\text{Reductive}\;\text{antioxidant}=\text{Fe}{{\left(\text{CN}\right)}_{6}}^{-4}\\ \text{Fe}{{\left(\text{CN}\right)}_{6}}^{-4}+{\text{FeCl}}_{3}⟶{\text{Fe}}_{4}{\left[\text{Fe}{\left(\text{CN}\right)}_{6}\right]}^{3}. \end{array}$$

Since FRAP assay is easy to perform and linearly related to molar concentration of the antioxidant present in it. Fe(III) reduction is used as an indicator of electron donating activity, which is due to its phenolic antioxidant action [31]. The FRAP values of extracts were calculated from the linear regression equation of the standard.

The result of phosphomolybdic acid method shows that extracts demonstrate electron donating capacity and thus transform free radical species into stable nonreactive products. FTC method is used to measure the amount of peroxide produced at the initial stage of lipid peroxide, whereas TBA method is used to measure the same at a later stage when peroxide decomposes to form carbonyl compounds. FTC and TBA values of butanol extract were found to be high as compared to the standard. It is observed from the UPLC analysis of both the extracts that butanol extract showed high antioxidant nature due to the presence of more polyphenols and in more concentration (Table 3) as compared to hexane extract.

## 5. Conclusions

To our knowledge, this is the first report demonstrating that butanol and hexane extracts of* P. aculeata* have antioxidant activity as seen in the DPPH, free radical assay, CUPRAC, non-site-specific and site-specific hydroxyl radical scavenging assays, FRAP, TAC, FTC, and TBA assay. Furthermore, our results showed that crude butanol extract of* P. aculeata* leaf might have good potential as a source for natural health products due to its antioxidant activities. The UPLC analysis of extracts showed that antioxidant nature of plant is due to the presence of these polyphenols. But, further studies are also needed for better clarifying the anticytotoxicity and various other biological properties of the plant species presented here.

## Figures and Tables

**Table 1:** The phytochemical components of the leaves of *Parkinsonia aculeata *Linn. based on the preliminary screening of extracts.

Serial number	Chemical tests	Butanol	Hexane
1	Alkaloid	+	−
2	Carbohydrate	+	+
3	Glycoside	−	−
4	Saponin	+	+
5	Protein and amino acids	+	−
6	Phenolic	+	+
7	Flavonoids	+	+

(+) indicates positive results; (−) indicates negative results.

**Table 2:** Ferric reducing antioxidant potential (FRAP) and total antioxidant capacity (TAC) activities of butanol and hexane leaves extracts of *P.  aculeata. *

Extracts	FRAP^a^	TAC^b^
Butanol	678 ± 0.23	36 ± 0.22
Hexane	60 ± 0.14	0.75 ± 0.06

Data represented as mean ± SE (*n* = 3).

^a^Expressed as *μ*M Fe (II)/g.

^b^Expressed as mg AAE/100 mg.

**Table 3:** Concentrations of different polyphenols present in the leaf extracts of *P. aculeata* Linn.

Serial number	Polyphenolic compounds	Concentrations	
		Butanol	Hexane
1	Gallic acid	0.594	—
2	Catechin	4.527	1.087
3	Chlorogenic acid	0.242	—
4	Epicatechin	0.794	—
5	Caffeic acid	21.223	0.237
6	Umbelliferone	214.679	3.463
7	Coumaric acid	21.552	0.477
8	Rutin	2.056	—
9	Ellagic acid	76.115	2.283
10	Tert-Butyl hydroquinone	248.134	5.254
11	Quercetin	—	0.586
12	Kaempferol	—	2.447

## References

[1] Muramatsu H, Kogawa K, Tanaka M (1995). Superoxide dismutase in SAS human tongue carcinoma cell line is a factor defining invasiveness and cell motility. *Cancer Research*.

[2] Steinberg D, Parthasarathy S, Carew TE, Khoo JC, Witztum JL (1989). Beyond cholesterol: modifications of low-density lipoprotein that increase its atherogenicity. *The New England Journal of Medicine*.

[3] Ammar RB, Sghaier MB, Boubaker J (2008). Antioxidant activity and inhibition of aflatoxin B1-, nifuroxazide-, and sodium azide-induced mutagenicity by extracts from *Rhamnus alaternus* L. *Chemico-Biological Interactions*.

[4] Cohly HHP, Taylor A, Angel MF, Salahudeen AK (1998). Effect of turmeric, turmerin and curcumin on H_2_O_2_-induced renal epithelial (LLC-PK1) cell injury. *Free Radical Biology and Medicine*.

[5] Shon M-Y, Kim T-H, Sung N-J (2003). Antioxidants and free radical scavenging activity of *Phellinus baumii* (*Phellinus of Hymenochaetaceae*) extracts. *Food Chemistry*.

[6] Zhang D, Yasuda T, Yu Y (1996). Ginseng extract scavenges hydroxyl radical and protects unsaturated fatty acids from decomposition caused by iron-mediated lipid peroxidation. *Free Radical Biology and Medicine*.

[7] Sala A, Del Carmen Recio M, Giner RM (2002). Anti-inflammatory and antioxidant properties of *Helichrysum italicum*. *Journal of Pharmacy and Pharmacology*.

[8] Veerapur VP, Prabhakar KR, Parihar VK (2009). *Ficus racemosa* stem bark extract: a potent antioxidant and a probable natural radioprotector. *Evidence-Based Complementary and Alternative Medicine*.

[9] Muselík J, García-Alonso M, Martín-López MP, Žemlička M, Rivas-Gonzalo JC (2007). Measurement of antioxidant activity of wine catechins, procyanidins, anthocyanins and pyranoanthocyanins. *International Journal of Molecular Sciences*.

[10] Besson E, Chopin J, Gunasegaran R, Nair AGR (1980). C-glycosylflavones from *Parkinsonia aculeata*. *Phytochemistry*.

[11] Chakraborty DD, Ravi V, Chakraborty P (2010). Phytochemical evaluation and TLC protocol of various extracts of *Bombax ceiba* Linn. *International Journal of Pharmaceutical Sciences and Research*.

[12] Singleton VL, Rossi JA (1965). Colorimetry of total phenolics with phosphomolybdic- phosphotungstic acid reagents. *American Journal of Enology and Viticulture*.

[13] Zhishen J, Mengcheng T, Jianming W (1999). The determination of flavonoid contents in mulberry and their scavenging effects on superoxide radicals. *Food Chemistry*.

[14] Blois MS (1958). Antioxidant determinations by the use of a stable free radical. *Nature*.

[15] Oyaizu M (1986). Studies on product of browning reaction prepared from glucose amine. *Journal of Nutrition*.

[16] Apak R, Güçlü K, Özyürek M, Karademir SE (2004). Novel total antioxidant capacity index for dietary polyphenols and vitamins C and E, using their cupric ion reducing capability in the presence of neocuproine: CUPRAC method. *Journal of Agricultural and Food Chemistry*.

[17] Halliwell B, Gutteridge JMC, Aruoma OI (1987). The deoxyribose method: a simple ’test-tube’ assay for determination of rate constants for reactions of hydroxyl radicals. *Analytical Biochemistry*.

[18] Rosen GM, Rauckman EJ (1984). Spin trapping of superoxide and hydroxyl radicals. *Methods in Enzymology*.

[19] Benzie IFF, Strain JJ (1996). The Ferric Reducing Ability of Plasma (FRAP) as a measure of ’antioxidant power’: the FRAP assay. *Analytical Biochemistry*.

[20] Prieto P, Pineda M, Aguilar M (1999). Spectrophotometric quantitation of antioxidant capacity through the formation of a phosphomolybdenum complex: specific application to the determination of vitamin E. *Analytical Biochemistry*.

[21] Kikuzaki H, Usuguchi J, Nakatani N (1991). Constituents of Zingiberaceae. I. Diarylheptanoids from the rhizomes of ginger (*Zingiber officinale* roscoe). *Chemical and Pharmaceutical Bulletin*.

[22] Kikuzaki H, Nakatani N (1993). Antioxidant effects of some ginger constituents. *Journal of Food Science*.

[23] Mruthunjaya K, Hukkeri VI (2008). In vitro antioxidant and free radical scavenging potential of *Parkinsonia aculeata* Linn. *Pharmacognosy Magazine*.

[24] Singh P, Shrivastava R, Saxena RC, Sharma M, Karchuli MS, Tripathi J (2011). Phytochemical screening and evaluation of antioxidant activity of *Parkinsonia aculeate* L. (Family-Leguminoseae) leaves extract. *International Journal of PharmTech Research*.

[25] Nanjo F, Goto K, Seto R, Suzuki M, Sakai M, Hara Y (1996). Scavenging effects of tea catechins and their derivatives on 1,1- diphenyl-2-picrylhydrazyl radical. *Free Radical Biology and Medicine*.

[26] Meir S, Kanner J, Akiri B, Philosoph-Hadas S (1995). Determination and involvement of aqueous reducing compounds in oxidative defense systems of various senescing leaves. *Journal of Agricultural and Food Chemistry*.

[27] Ferreira ICFR, Baptista P, Vilas-Boas M, Barros L (2007). Free-radical scavenging capacity and reducing power of wild edible mushrooms from northeast Portugal: individual cap and stipe activity. *Food Chemistry*.

[28] Hazra B, Biswas S, Mandal N (2008). Antioxidant and free radical scavenging activity of *Spondias pinnata*. *BMC Complementary and Alternative Medicine*.

[29] Sharma S, Vig AP (2013). Evaluation of *in vitro* antioxidant properties of methanol and aqueous extracts of *Parkinsonia aculeata* L. leaves. *The Scientific World Journal*.

[30] Griffin SP, Bhagooli R (2004). Measuring antioxidant potential in corals using the FRAP assay. *Journal of Experimental Marine Biology and Ecology*.

[31] Buyukokuroglu ME, Gulcin I, Oktay M, Kufrevioglu OI (2001). *In vitro* antioxidant properties of dantrolene sodium. *Pharmacology Research*.

